# A process evaluation of how the routine vaccination programme is implemented at GP practices in England

**DOI:** 10.1186/s13012-018-0824-8

**Published:** 2018-10-22

**Authors:** Tim Crocker-Buque, Michael Edelstein, Sandra Mounier-Jack

**Affiliations:** 1 Faculty of Public Health and Policy, London School of Hygiene and Tropical Medicine, 15-17 Tavistock Place, London, WC1H 9SH UK; 2 Department of Immunisation, Hepatitis and Blood Safety, Public Health England, 61 Colindale Avenue, London, NW9 5EQ UK

**Keywords:** Vaccination, Immunisation, Implementation, Health service, Primary care

## Abstract

**Background:**

In recent years, the incidence of several pathogens of public health importance (measles, mumps, pertussis and rubella) has increased in Europe, leading to outbreaks. This has included England, where GP practices implement the vaccination programme based on government guidance. However, there has been no study of how implementation takes place, which makes it difficult to identify organisational variation and thus limits the ability to recommend interventions to improve coverage. The aim of this study is to undertake a comparative process evaluation of the implementation of the routine vaccination programme at GP practices in England.

**Methods:**

We recruited a sample of geographically and demographically diverse GP practices through a national research network and collected quantitative and qualitative data as part of a Time-Driven Activity-Based Costing analysis between May 2017 and February 2018. We conducted semi-structured interviews with practice staff involved in vaccination, who then completed an activity log for 2 weeks. Interviews were transcribed and coded using a framework method.

**Results:**

Nine practices completed data collection from diverse geographic and socio-economic contexts, and 52 clinical and non-clinical staff participated in 26 interviews. Information relating to 372 vaccination appointments (233 childhood and 139 adult appointments) was captured using activity logs. We have defined a 14-stage care delivery value chain and detailed process map for vaccination. Areas of greatest variation include the method of reminder and recall activities, structure of vaccination appointments and task allocation between staff groups. For childhood vaccination, mean appointment length was 15.9 min (range 9.0–22.0 min) and 10.9 min for adults (range 6.8–14.1 min). Non-clinical administrative activities comprised 59.7% total activity (range 48.4–67.0%). Appointment length and total time were not related to coverage, whereas capacity in terms of appointments per eligible patient may improve coverage. Administrative tasks had lower fidelity of implementation.

**Conclusions:**

There is variation in how GP practices in England implement the delivery of the routine vaccination programme. Further work is required to evaluate capacity factors in a wider range of practices, alongside other contextual factors, including the working culture within practices.

## Background

The introduction of vaccinations into public health programmes in European countries over the last century has resulted in a dramatic reduction in vaccine preventable diseases (VPDs), including the elimination of smallpox and polio from the region. However, in recent years, the incidence of several pathogens of public health importance such as measles, mumps, pertussis and rubella has increased in Europe [1]. Coverage of the combined measles, mumps and rubella vaccine (MMR) is below herd immunity thresholds in many European countries, and in 2018, several countries such as Italy, Greece, France, Romania and the UK were affected by outbreaks [2, 3]. The causes of disparities in coverage are complex, multifactorial and country-specific but include suboptimal delivery mechanisms, alongside inequities in access for some populations, particularly those living in low-income urban environments [1, 4, 5].

In England, primary care clinics (GP practices) are responsible for arranging most routine childhood and adult vaccinations for their local population. The regular addition of antigens into routine vaccine programmes has meant organising service delivery has become increasingly complex [6]. In 1990, the programme consisted of eight vaccinations for children and adolescents against diphtheria, pertussis, tetanus, polio, measles, mumps, rubella and tuberculosis [7]. The programme for 2016–2017 (study period) and changes for 2018 are presented in Table 1 and now contains 16 childhood, 2 adolescent and 3 adult vaccinations, as well as 2 in pregnancy and a wide range for people with medical co-morbidities [8].

**Table 1:** The 2016–2017 routine vaccine programme in England, adapted from Public Health England (2018) [8]

Age	Disease(s)	Vaccine (trade name)	Notes, including schedule changes implemented during the study period
8 weeks	Diphtheria, tetanus, pertussis, polio, Haemophilus influenza type b (Hib)	DTaP/IPV/Hib (Pediacel or Infanrix IPV Hib)	Changed to Infanrix Hexa, with hepatitis B (Hep B) included in 2018
	Pneumococcal (13 serotypes)	Pneumococcal Conjugate (PCV) (Prevenar 13)	
	Meningococcal group B (MenB)	MenB (Bexsero)	
	Rotavirus	Rotavirus (Rotarix)	
12 weeks	Diphtheria, tetanus, pertussis, polio and Hib	DTaP/IPV/Hib (Pediacel or Infanrix IPV Hib)	Changed to Infanrix Hexa, with hepatitis B (Hep B) included in 2018
	Rotavirus	Rotavirus (Rotarix)	
16 weeks	Diphtheria, tetanus, pertussis, polio and Hib	DTaP/IPV/Hib (Pediacel or Infanrix IPV Hib)	Changed to Infanrix Hexa, with hepatitis B (Hep B) included in 2018
	MenB	MenB (Bexsero)	
	Pneumococcal (13 serotypes)	PCV (Prevenar 13)	
1 year	Hib and MenC	Hib/MenC booster (Menitorix)	
	Pneumococcal 13	PCV Booster (Prevenar 13)	
	Measles, mumps and rubella (MMR)	MMR (VaxPRO or Priorix)	
	MenB	MenB booster (Bexsero)	
2–6 years	Influenza (seasonal)	Live attenuated influenza vaccine (LAIV) (Fluenz Tetra)	Seasonal vaccine—excluded from this study
From 3 years 4 months	Diphtheria, tetanus, pertussis and polio	DTaP/IPV (Infanrix IPV or Repevax)	
	MMR	MMR (VaxPRO or Priorix)	
Females 12–13 years	Human papillomavirus (HPV)	HPV (two doses 6 to 24 months separated) (Gardasil)	Usually given in school—excluded from this study unless given in the GP practice
14 years	Tetanus, diphtheria and polio	Td/IPV (Revaxis)	Usually given in school—excluded from this study unless given in the GP practice
	Meningococcal groups A, C, W and Y	Men ACWY (Nimenix or Menveo)	Usually given in school—excluded from this study unless given in the GP practice
65 years	Pneumococcal (23 serotypes)	Pneumococcal polysaccharide (PPV) (Pneumovax II)	
> 65 years	Influenza (seasonal)	Inactivated influenza vaccine (strain dependent)	Seasonal vaccine—excluded from this study
70 years	Shingles	Shingles (Zostavax)	
Condition	Disease(s)	Vaccine (trade name)	Notes
Pregnancy	Influenza (seasonal)	Inactivated influenza vaccine (strain dependent)	Seasonal vaccine—excluded from this study
	Pertussis	DTaP/IPV (Boostrix-IPV)	From 16 weeks gestation
Various underlying medical conditions	Meningococcal Pneumococcal Influenza Hepatitis A and B HiB	Various	Patients with a wide range of medical conditions (asplenia, diabetes, respiratory, neurological) are eligible for a range of vaccines

Delivering these vaccinations has required an increase in the number of contacts needed with the healthcare system for a wider range of patients, alongside associated administrative activities, including reminding and recalling patients and collecting and submitting data. These activities add significantly to the cost base of a practice and have taken place contemporaneously with increasing demand for GP services from an ageing population with more complex health needs, without commensurate increases in funding or staffing [9]. In addition, the public health system in England underwent a dramatic reorganisation in 2013 following the implementation of the Health and Social Care Act 2012. This moved local public health functions from NHS providers into local government, created a new executive agency for public health within the Department of Health called Public Health England and moved commissioning of public health services to a new agency called NHS England, with the aim of putting ‘clinicians at the centre of commissioning, free[ing] up providers to innovate, empower[ing] patients and giv[ing] a new focus to public health’ [10]. However, this had unintended consequences for the vaccination programme, resulting in a ‘complex mesh’ of ‘fractured’ commissioning, policy and service provision organisations and reduced oversight and support [11].

**Fig. 1:**
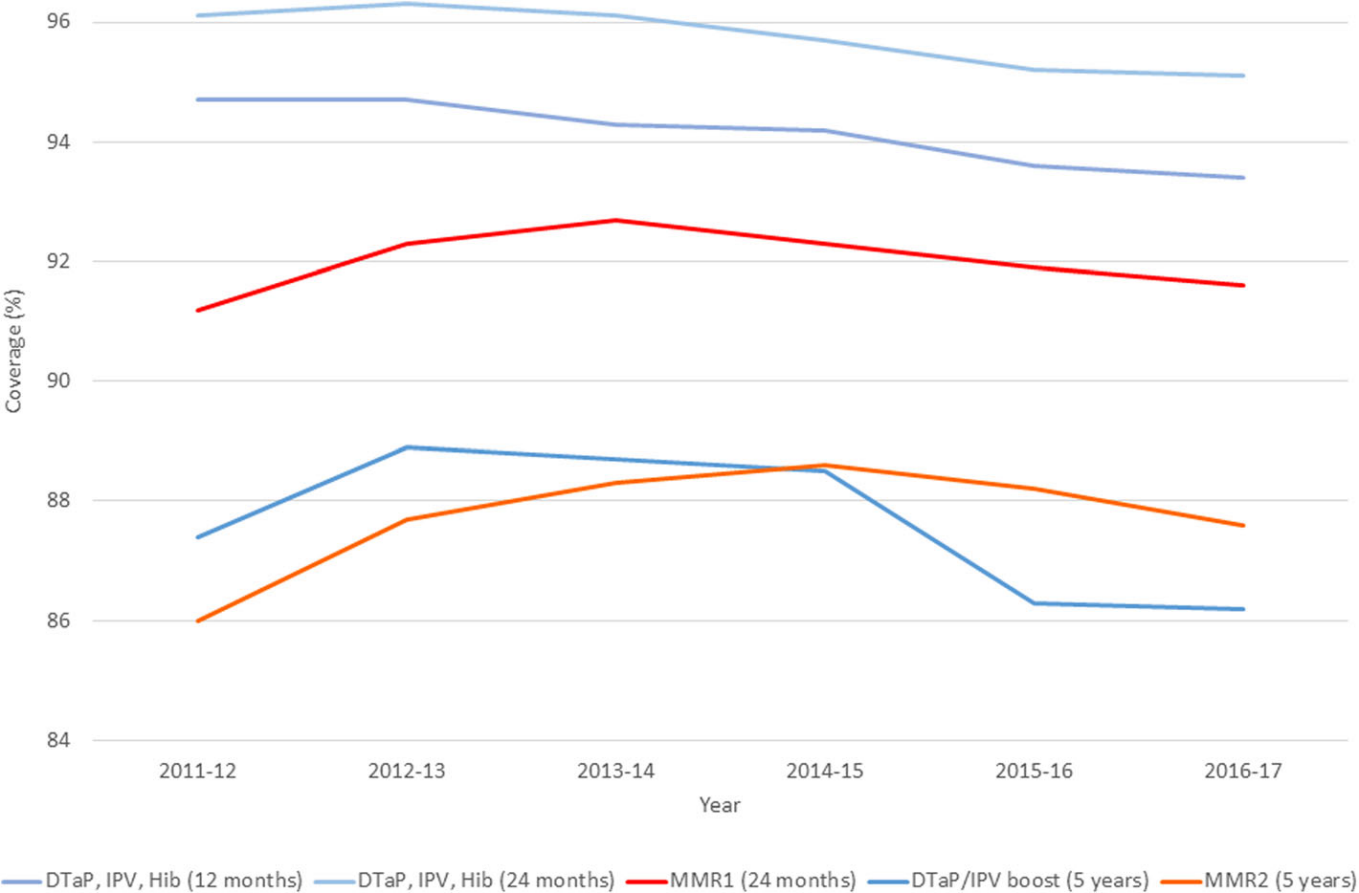
Coverage (%) of selected vaccinations by age in England 2011–2017, from NHS Digital (2017) [11]

Vaccination is a highly complex public health programme with multiple interdependent activities which currently exhibits divergent outcomes [12]. Routine coverage statistics derived from GP practice records show that while coverage for childhood vaccinations remains relatively high, there have been multi-year decreases in coverage across many vaccines (Fig. 1) [13]. While this could be a result of expected fluctuations, coverage had previously been on an upward trend for most vaccines over the preceding decade. Further year-on-year decreases may result in coverage for some vaccines reducing below international targets (> 95% coverage) and population immunity thresholds to prevent outbreaks (> 90% coverage). For example, MMR at 24 months increased every year between 2007-2008 and 2012-2013 and, however, has now reduced from a high of 92.7% (2013–2014) to 91.6%. The decreases shown for the DTaP/IPV booster and MMR at 5 years may mean the > 90% target will not now be reached. This is particularly concerning for the measles-containing vaccines in the context of current large outbreaks in several European countries and five UK locations [2, 3].

National averages also hide significant geographic inequities. For example, coverage is significantly lower in London than elsewhere in England (e.g. in 2016–2017 DTaP-IPV-Hib 12 months: England 93.4%, London 88.8%; MMR2 5 years: England 87.6%, London 79.5%). There is also lower coverage in some ethnic groups and in areas of higher deprivation [14, 15]. The causes for this are unclear at present, and this study aims to provide more information about what is happening at GP practice level.

Previous research has shown that lower adherence to programme components is associated with worse outcomes for prevention services [16]. GP practices are independent providers contracted to provide vaccine services by NHS England, which defines minimum service standards for vaccination in the core service specification [17]. The specification leaves significant autonomy to practices as to how they organise implementing the programme. The National Institute for Health and Care Excellence (NICE) has also published quality standards for aspects of vaccination programme delivery for children and adolescents [18]. However, it is currently not known how different practices implement the programme, i.e. how activities are managed, nor whether the quality standards are adhered to. This makes it difficult to identify organisational variation and thus limits the ability to recommend interventions to improve coverage. Therefore, the aim of this study is to undertake a comparative process evaluation of the implementation of the routine vaccination programme at GP practices in England.

## Methods

We collected quantitative and qualitative process data in line with the Medical Research Council’s process evaluation guidance as part of Time-Driven Activity-Based Costing (TDABC) analysis [12]. TDABC is a method for allocating costs to complex processes within organisations to identify cost drivers and opportunities for lowering costs while improving quality [19–21], which has been extensively applied to complex health service delivery in primary and secondary care [21]. Steps 1–4 of the 7-step TDABC analysis generate process evaluation data [22]. Steps 5–7 involve calculation and allocation of costs and are excluded from this analysis. Qualitative components of this study have been reported in line with COREQ guidelines [23].

- Step 1: Define the condition and population

The condition under analysis is a patient registered at a GP practice who is eligible for a routine vaccination. The organisational boundary of this analysis is those activities that are undertaken within the GP practice by employees. Patients attending for all routine childhood and adult vaccinations were included (Table 1), except seasonal influenza, as this is funded through a different mechanism and activity is uneven throughout the year. Non-routine vaccines (e.g. travel vaccinations) and vaccines not given in GP practices (e.g. HPV vaccine given at school) were also excluded.

- Steps 2 and 3: Define the delivery value chain and develop a process map

The care delivery value chain (CDVC) is a visual representation of the main activities involved in providing routine vaccinations at GP practices [21, 22]. To define the CDVC, we conducted semi-structured interviews using primarily open questions with a selection of staff from each practice (individually or in groups depending on availability) using a pre-defined topic guide (Appendix). One author (TCB) conducted all the interviews during a single day at each practice, which were recorded, transcribed verbatim and uploaded into NVIVO v11 for analysis. Contemporaneous field notes were made during site visits. Coding was completed by one author (TCB) using a framework method. A deductive code tree was constructed from core process evaluation elements (inputs, processes, activities, outputs, outcomes and impact) as well as temporal associations within the implementation process (pre-appointment, appointment, post-appointment) and age-group codes (children and adults). Inductive codes were used to identify important issues arising from the data (e.g. funding, outbreaks, appointment length). During the first pass, we coded all data using deductive codes and generated the inductive codes. We then undertook a second pass to universally apply the inductive codes and check completeness and overall accuracy. The framework and a selection of transcripts were reviewed by a second author (SMJ) [24].

- Step 4: Allocate time estimates for each process step

We provided all staff involved in vaccination at each practice an activity log to contemporaneously record all clinical and non-clinical vaccination activity over a 2-week period. We modified logs used in a study conducted in New Zealand [25] and validated them with practice managers (PMs), practice nurses (PNs) and GPs at two non-participating practices. The first practice recruited was used as a pilot before data collection tools were finalised following minor changes to the interview guide and to the data collection tools, primary for clarity and usability. Following the interviews (step 2), a list of every member of staff involved in vaccination was developed for each practice who were then trained and provided with an activity log to keep for 2 weeks. Data were extracted and placed into a Microsoft Excel file for analysis.

### Sampling

We recruited GP practices to participate through the National Institute for Health Research Clinical Research Network (CRN) [26]. This comprises an extensive network of primary and secondary care organisations in all regions of England, including more than 2000 GP practices, who apply to join a scheme of payment for participating in research projects funded by the Department of Health. We aimed to recruit a non-representative convenience sample of 10 practices (due to our available capacity) from a range of geographic and socio-economic contexts. Following circulation of the study details, 14 practices retuned expressions of interest to participate over a 9-month period before recruitment was ended. We excluded 4 practices as they were geographically similar to already included practices, and 1 did not complete data collection. A £350 shopping voucher (£500 in London) was provided to participating practices as compensation for staff time. Agreement to participate was made with the practice manager, who we then asked to identify relevant members of staff to participate in interviews to explore the organisation of vaccination within the practice. Due to the significant variation in size, staff profile and administrative organisation between practices, we specified only that the practice manager and a practice nurse must be included, and the manager was then free to recruit other relevant staff to participate based on the research aims.

### Consent and ethical approval

We gained written, informed consent from each participating staff member prior to commencing data collection. No patients were involved. The study received ethical approval from the LSHTM Ethics Committee and the NHS Health Research Authority (project ID 212278).

## Results

Nine practices completed data collection activities between May 2017 and February 2018. Their characteristics are presented in Table 2, which demonstrate wide geographic and demographic diversity. Two quality metrics are routinely collected and reported at GP practice level: Quality Outcomes Framework (QOF), a pay for performance scheme in which vaccination is not included (except influenza coverage in people with medical co-morbidities), and the proportion of patients recommending the practice to friends and family. All practices aside from C and J score higher QOF points than average, and every practice scores higher on the friends and family test, except F, which scores very low (56.6%). For childhood vaccination coverage, the smaller practices have higher than average coverage for most vaccinations (A, B, C, D, E and F) whereas the three largest practices (G, H and J), including the two in London, have lower coverage, with practice G having the lowest coverage overall. Adult vaccinations are similar with the smaller practices scoring above average and the four larger practices (F, G, H and J) below with particularly low coverage in practice J.

**Table 2:** Characteristics of GP practices included in the study

	England average	A	B	C	D	E	F	G	H	J
Region	–	East Midlands	East of England	Yorks and Humber	South West	East Midlands	East of England	North East and Central London	South East	South London
Urban/rural status^a^	–	Mainly rural	Urban, city and town	Urban, city and town	Mainly rural	Urban, city and town	Largely rural	Major conurbation	Largely rural	Major conurbation
List size^b^	7000	4600	6600	7000	8100	12,600	13,800	14,000	16,000	20,000
Demography										
Deprivation decile^b^	–	8	2	1	8	10	7	4	6	4
Minority ethnic groups (%)^b^	–	1.6	6.2	12.0	1.3	1.8	3.4	30.3	2.1	41.4
Aged 0–4 years (%)^b^	5.7	3.3	7.3	5.7	4.4	4.6	5.0	5.0	5.4	7.7
Aged 65+ years (%)^b^	17.3	31.6	10.4	13.6	30.4	23.6	21.5	11.3	18.9	11.8
Quality indicators										
QOF achievement (%)^b^	95.6	99.6	98.2	94.5	98.9	99.3	96.2	95.7	99.6	93.7
Patients recommending practice (%)^b^	77.4	95.3	78.4	81.4	89.3	84.4	56.6	87.8	83.1	85.9
Childhood vaccination coverage										
DTP-IPV-Hib 3 doses by 12 months (%)^c^	93.4	98.9	96.0	97.3	96.0	98.9	98.7	78.7	90.6	91.2
DTP-IPV-Hib 3 at 24 months (%)^c^	95.1	100.0	95.8	100.0	97.5	100.0	98.1	91.4	90.8	94.6
MMR 1 by 24 months (%)^c^	91.6	100.0	97.9	98.6	93.7	97.2	97.5	78.1	85.5	86.9
MMR 2 by 5 years (%)^c^	87.6	94.4	94.2	95.5	98.3	94.4	93.1	69.6	85.1	88.4
MMR 2 by 5 years (%)^d^	83.4	100.0	92.7	93.0	98.8	96.8	93.8	79.7	–	74.5
Adult vaccination coverage										
PPV (%) 2017–2018, 70–74^d^	70.2	79.0	71.3	81.9	83.3	88.7	56.1	64.4	65.6	42.9

*QOF* Quality Outcomes Framework, *DTP-IPB-Hib 3* Diphtheria, tetanus, pertussis, polio and haemophilus influenzae group b, 3rd dose, *MMR* measles, mumps and rubella vaccine, *PPV* pneumococcal polysaccharide vaccine

Data sources: ^a^2011 Rural-Urban Classification of Local Authorities (https://www.gov.uk/government/statistics/2011-rural-urban-classification-of-local-authority-and-other-higher-level-geographies-for-statistical-purposes) [25]; ^b^National General Practice Profiles; for deprivation, 10 is most deprived decile and 1 is least deprived (https://fingertips.phe.org.uk/profile/general-practice) [26]; ^c^derived from UNIFY 2 data 2016–2017, which are experimental management data and have lower reliability [11]; ^d^derived from Immform data 2016–2017

### Care delivery value chain and process map

In total, 52 people participated in 26 interviews at the 9 practices. Interviews ranged from 10 to 75 min. The number and type of staff who participated at each practice is presented in Table 3. In one practice, a GP provided oversight to the programme out of personal interest (J), but otherwise, no doctors were involved in vaccination.

**Table 3:** Number and type of staff group participating in semi-structured interviews at each practice

	A	B	C	D	E	F	G	H	J	TOTAL
Practice nurse (PN)	2	2	2	2	6	1	3	2	3	23
Healthcare assistant (HCA)	0	0	0	0	4	0	1	2	0	7
Practice manager (PM)	1	1	1	1	1	1	1	0	1	8
Administrator (AD)	0	1	2	1	2	1	1	3	1	12
Receptionist (R)	0	0	1	0	0	0	1	0	0	2
Total	3	4	6	4	13	3	7	7	5	52

**Fig. 2:**
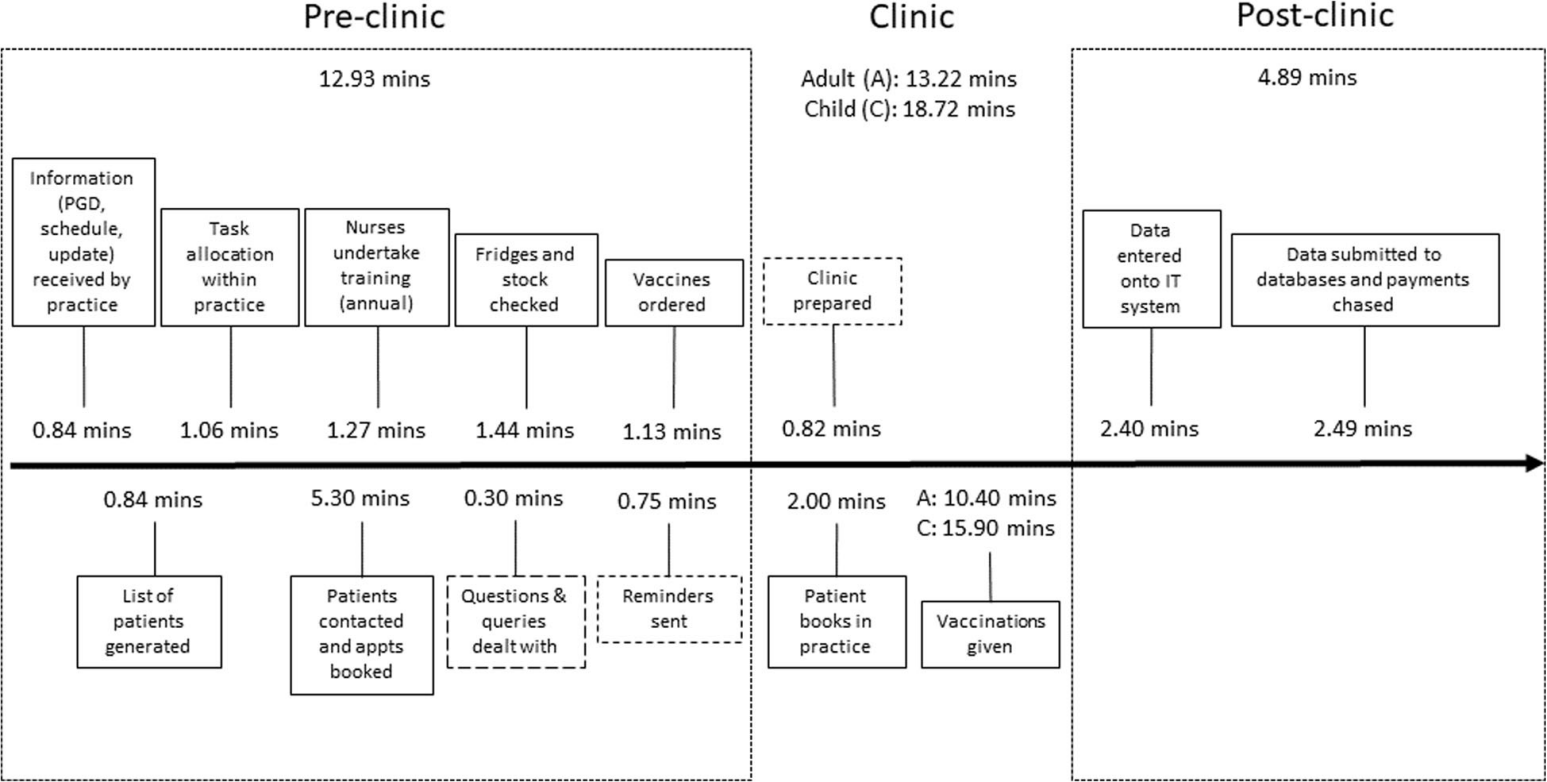
The care delivery value chain for routine vaccinations from the perspective of a primary care practice. (The black arrow shows the process over time within the GP practice, with activities involving the practice above the line and patient-facing activities below the line. The blue arrow represents the patient’s interaction with the practice during the process). Activity steps with dashed outline are those where times were sometimes recorded together with other activities by practices, so timings are less reliable. Timings are mean times calculated across the included practices for each step

**Fig. 3:**
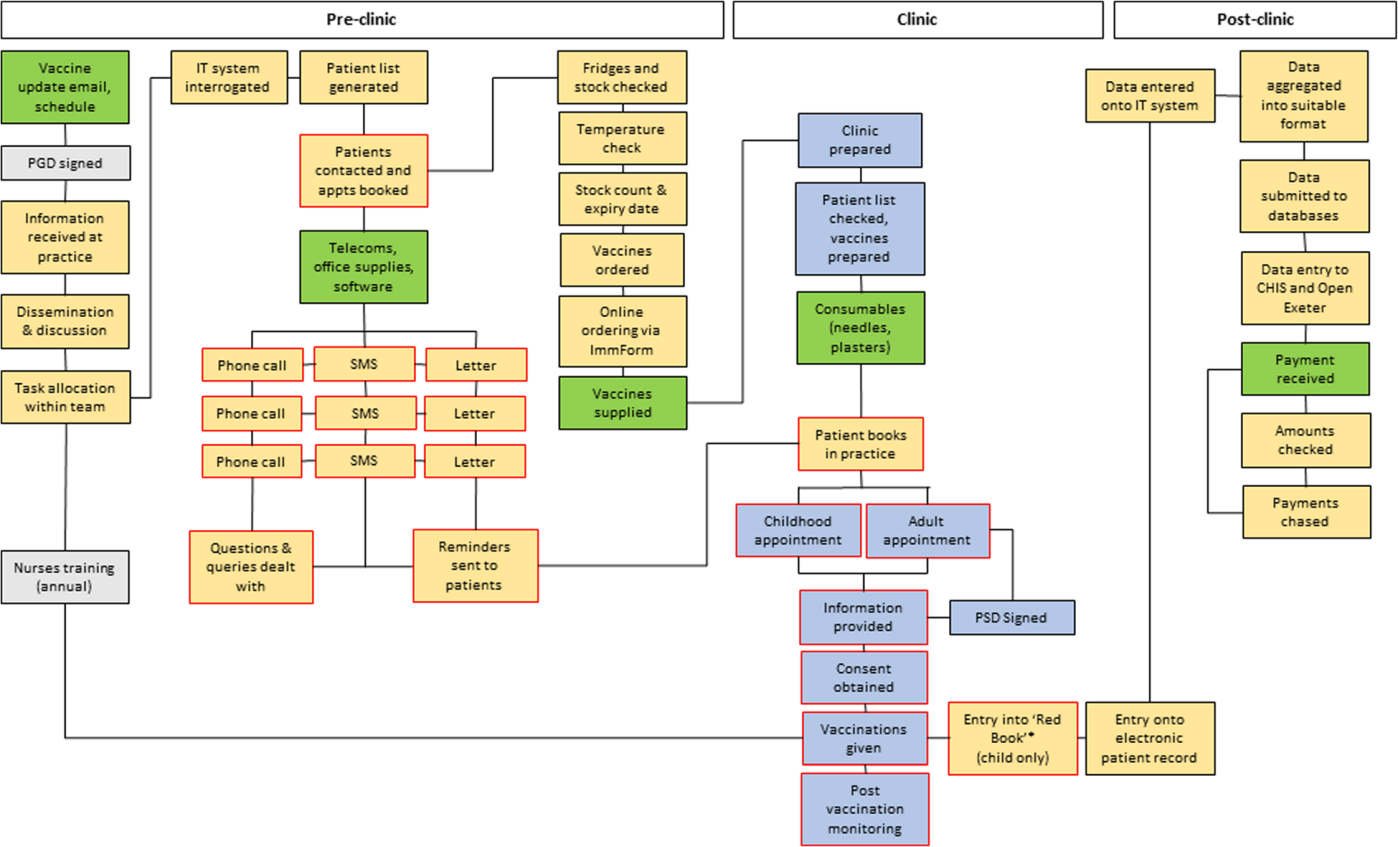
A process map of the system of implementing routine vaccination at GP practices in England. Green box = resources; yellow box = non-clinical/administrative activities and processes; grey box = activity undertaken off GP practice site; blue box = clinical activity and processes; red outlines = activities and process with direct patient contact. *The Red Book is a paper health record held by a child’s carers. PGD patient group directive, SMS text message (short message service), PSD patient-specific directive; CHIS Child Health Information System

Interview data were supplemented with the activity log data to confirm details provided in the interviews, and information relating to 372 vaccination appointments was captured, comprising 233 childhood and 139 adult appointments. The resulting CDVC is presented in Fig. 2. Fourteen core activities were common to all practices and took place within the GP practice building, aside from the nurses’ annual training, which was usually at another local organisation. Timings for three of the activities (dashed boxes) were aggregated with other activities by some practices. Although this is presented as a linear process, these activities often take place concurrently or non-sequentially creating a constant responsive workflow. A process map from the perspective of the practice is presented in Fig. 3.

### Activities and task allocation

The task allocation of the activities shown in Figs. 2 and 3 is presented in Table 4. The role of the clinical staff was similar across practices, with practice nurses (PNs) having the primary role in delivering vaccinations, with some also delivered to adults by healthcare assistants (HCAs), who work under the guidance of the nurses. However, the roles for administrative staff varied widely, with some practice managers (PMs) having an oversight role and some being directly involved in activities such as reminder/recall; some practices had general administrators with various roles in managing reminder/recall activities, following up patients, planning clinics and submitting data, while others employed specialist administrators with specific roles, such as data management, target focussed roles or managing aspects of finance. In other practices, an assistant practice manager (APM) filled this role. Most receptionists had a role booking in appointments and checking patients in on arrival, but in some practices, they had a large role in reminder/recall activities and counselling patients over the phone.

**Table 4:** Organisation of responsibilities for processes involved in routine vaccination at the included practices

Activities	A	B	C	D	E	F	G	H	J
1. Pre-clinic administration (receiving info; generating patient list)	The PM runs searches and allocates tasks to the R staff.	An AD runs the searches and manages the list.	The PM runs searches and the PN has a role in allocating tasks.	The PM runs searches and allocates tasks to the R staff.	The APM is responsible for running searches and allocating tasks.	The PM runs the searches and then tasks allocated to the PN and AD.	An AD generates the list and allocates tasks, with some support from the PN.	An AD generates the lists of eligible patients, from info from CHIS and local records.	An AD is responsible for generating the list of patients.
2. Patient contact, appointment booking and reminder/recall activities	The R staff are responsible for booking appointments and reminder recall activities, which involve letters and phone and text messages. Occasionally the PN will phone parents.	An AD is then responsible for all the reminder and recall activities, which primarily involves letters, but with some follow-up phone calls.	An AD has a large role in contacting patients, including calling patients and sending letters and SMS reminders, with some support from a R.	The R staff undertake all appointment booking and reminder recall activities and have a role in clinic preparation. Initial invite is by letter, followed by phone calls. Adult vacc is opportunistic.	This is split between the APM and the R staff and primarily involves sending letters, with some follow-up phone calls.	Some initial letters sent, then an AD books appts during the PN’s clinic (below). The PN and PM both also have a role here in phoning patients who do not respond or DNA.	The R staff are responsible for sending out letters and SMS reminders and booking appts. An AD and PN also call parents who DNA or do not respond.	This is undertaken by an AD who sends letters to parents, or will phone or send a SMS in the case of no response. Adult vaccines are booked ad hoc, especially during flu season.	The AD also sends the letters and manages the reminder recall activities, and the PN phones non-responders and DNAs.
3. Vaccine ordering, stocking and fridge maintenance	The PN primarily manages vaccine stock levels although with some support from the PM.	An HCA undertakes the vaccine ordering, which stock audit, and fridge maintenance is done by the PN.	Ordering is done by the PN and stocking and maintenance is split between the PN and an AD.	Ordering, stock management and maintenance is undertaken by a PN.	The PN does the fridge maintenance and an AD vaccine stocking and ordering.	Vaccine ordering and fridge maintenance are all undertaken by the PN.	Stocking and ordering is undertaken by an AD with some maintenance from the PN.	Stocking and ordering is done by an AD. Fridge maintenance is split between an AD, a PN and HCA.	Stocking, ordering and maintenance are all undertaken by a PN.
4. Vaccination appointments	Vacc takes place in general clinics with a PN. Childhood vaccines are allocated 20 min, with some adult vaccs having 10-min appts.	All vacc takes place in general clinics within 15-min appts with a PN.	Most primary imms are given in a specific baby clinic with a PN in a 15-min appt, although some are also done in general clinics. All adult imms are done in general clinics and sometimes 10-min appts are used.	Almost all vacc takes place in a dedicated baby/child clinic with 10-min appts. Although rarely 20-min general appts are used.	A mixture of 15- and 20-min appts are used in general clinics with a PN. Rarely 10-min appts are used.	There is a dedicated clinic for the 8-week appts with 2 PNs working 5 min per patient. All the others in general clinics with 10-min appt with a PN.	All childhood and most adult vacc take place in 15-min appts with a PN. Some PCV and shingles vacc are done in 20-min appts with an HCA.	A specific vacc clinic is run that involves 2 PNs with 5-min appts and 2 HCAs undertaking data input. A few 15-min general appts with PN and 20-min appts with an HCA are used. Adult vaccs take place in general clinics and are often given by HCAs.	Almost all vacc are given in 15-min appts in a general clinic with a PN.
5. Post-clinic data collection and submission, including Open Exeter and CQRS.	This is entirely undertaken by the PM.	This is primarily done by the AD, with some support from the PM.	Some data collection is undertaken by the PN immediately after the appts, then an AD is responsible for upload and submission.	This task is split between the PM and the R staff, with a small amount of support from the PN.	This is undertaken by an AD staff member and however is primarily automated.	An AD is in the clinic with the PN to undertake data collection and submission simultaneously. The PM has a role in the financial submissions.	A member of the R team has a large role in this, supported by a specialist AD.	Aside from the data entry undertaken in the clinics, the remainder is done by the AD.	An AD has a large role in data submission, with some time spent by the PN, particularly on CQRS.
6. Professional tasks and activities (training, reading updates, PGD administration)	Reading the vaccine update and training are undertaken by the PN.	Reading the vaccine uptake is done by both the PM and PN, with the PN also doing training.	Reading the vaccine update and training are undertaken by the PN.	Reading the vaccine update and training are undertaken by the PN.	Reading the vaccine update and training are undertaken by the PN.	Reading the vaccine update and training are undertaken by the PN.	Reading the vaccine update and training are undertaken by the PN.	The PN undertakes annual training.	The PN undertakes annual training.

*PM* practice manager, *APM* assistant practice manager, *PN* practice nurse, *AD* administrator, *R* receptionist, *DNA* did not attend, *HCA* healthcare assistant, *PCV* pneumococcal vaccine, *SMS* text message

The two components that had the greatest variation between practices were (i) the mechanism and frequency of reminder/recall activities (Table 4, row 2) and (ii) the structure of the vaccination appointments, which varied in length and distribution throughout the week (Table 4, row 4). Several practices had specific clinics for vaccination: C (two clinics weekly, one for babies and one for older children), D and H (one weekly clinic for babies and children) and F (weekly baby clinic only). Elsewhere appointments were diffused throughout the week.

### Outputs

The number of childhood vaccination appointments (Table 1) ranged from 9 to 71 during the 2-week data collection periods (Table 5). The mean appointment length across all practices was 15.9 min (range 9.0–22.0 min). Smaller practices (A and C) had the longest appointments, and the shortest were observed in F and H both medium-large practices in rural areas. We asked clinical staff to add information on any other issues occurring in the appointment that could have impacted on time, and no appointments were identified that had a significant non-vaccination-related component.

**Table 5:** Data derived from the number and length of vaccination appointments and time spent on vaccination during the 10-day study period at each practice

	A	B	C	D	E	F	G	H	J
Childhood appointments (*n*)	9	14	15	23	24	30	23	31	71
Mean length, min (95% CI)	20.2 (18.2–22.2)	15.4 (13.1–17.6)	22.0 (16.4–27.6)	13.9 (11.5–16.3)	18.1 (16.0–20.1)	9.8 (8.9–10.7)	18.3 (15.5–21.1)	9.0 (8.0–10.5)	16.7 (15.4–17.8)
Annual appts per child 0–4 (*n*)	1.53	0.76	1.01	1.56	1.28	1.01	0.81	1.20	1.24
Adult appointments (*n*)	26	10	22	17	4	23	7	14	16
Mean length, min (95% CI)	9.2 (8.6–9.8)	11.2 (8.7–13.7)	14.1 (12.6–15.6)	8.7 (6.9–10.5)	6.8 (2.9–10.6)	8.0 (6.8–9.2)	13.3 (11.4–15.1)	9.1 (7.6–10.6)	13.5 (11.9–15.1)
Annual appts per adult aged 65+ (*n*)	0.45	0.38	0.57	0.17	0.04	0.19	0.11	0.11	0.17
Total time spent on vaccination during study period (TPVT) (min)	1205	915	1821	1017	973	1224	1481	1231	2711
Proportion of time spent on non-clinical tasks, min (%)	784 (65.1)	588 (64.3)	1180 (64.8)	550 (54.1)	512 (52.6)	746 (60.9)	893 (60.3)	825 (67.0)	1312 (48.4)
Proportion of time spent on clinical tasks, min (%)	421 (34.9)	327 (35.7)	641 (35.2)	467 (45.9)	461 (47.4)	478 (39.1)	588 (39.7)	406 (33.0)	1399 (51.6)
Relative time per patient (mean time per patient on list = 1)	1.86	0.98	1.84	0.89	0.55	0.63	0.75	0.55	0.96

To estimate the capacity available for vaccination, the number of children aged 0–4 was divided by an estimate of the annual number of child appointments (number during the study period multiplied by 25, equal to 50 working weeks). Practices A and D had the highest number (1.53 and 1.56 appts/child respectively) and are small-medium practices in rural areas of low deprivation with high coverage (Table 2). Practice B had relatively lower capacity (0.76 appts/child) and slightly lower coverage. Practice G (large practice in London) also had lower capacity (0.81 appts/child) and significantly lower coverage, whereas practice J (large practice with high number of registered children in London) had higher capacity (1.24 appts/child) and somewhat higher coverage overall.

The number of adult vaccination appointments ranged from 4 to 26 and are not related to practice size. The high number of adult vaccination appointments at practice A was due to a concurrent shingles campaign (23 of 26 appointments), and practice C was running a meningococcal ACWY campaign for adolescents (18 of 22 appointments). Appointment capacity was estimated by dividing the number of adults aged 65+ by an estimate of the annual number of adult appointments. This ranged from 0.04 (practice E) to 0.57 (practice C) and was not related to overall performance. This is likely due to the large number of PPV vaccinations delivered alongside the seasonal flu campaign, which has been excluded from this analysis. Observed appointment length ranged from 6.8 min (E) to 14.1 min (C), with the mean being 10.4 min. Overall mean time spent across the nine practices for each stage in the CDVC is presented in Fig. 2.

The mean proportion of total practice vaccination time (TPVT) spent on non-clinical activities was 59.7% (range 48.4–67.0%). At most practices (A, B, C, F, G, H), around two thirds TPVT was spent on non-clinical work, and in the remainder (D, E, J), it was split evenly. Practice J was the only location where clinical time (51.9%) outweighed non-clinical time due to the large number of childhood appointments.

**Fig. 4:**
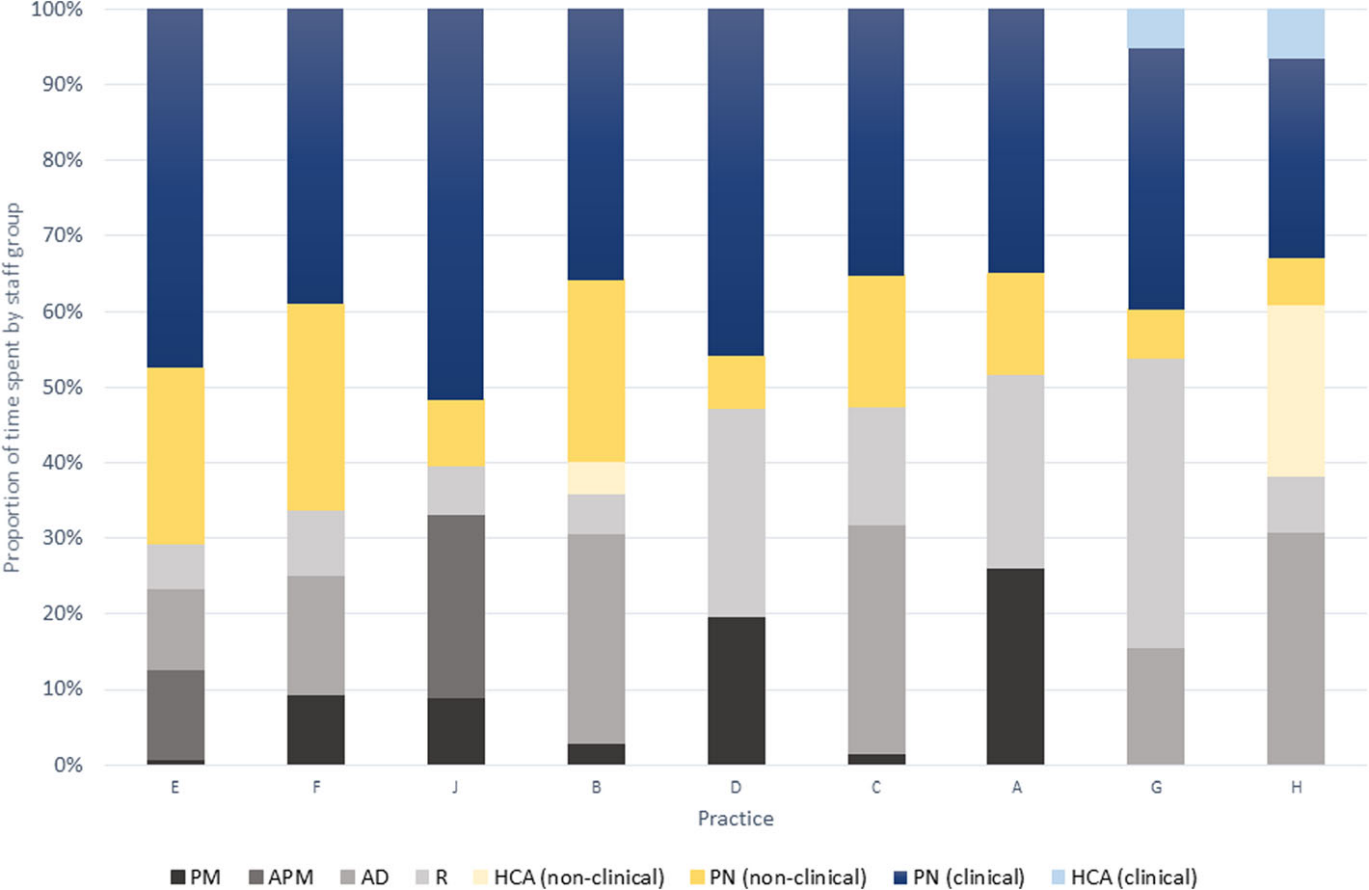
Proportion (%) of total practice vaccination time spent by different staff groups on clinical and non-clinical tasks (PM = practice manager; APM = assistant practice manager; PN = practice nurse; AD = administrator; R = receptionist; HCA = healthcare assistant)

To account for practice size, TPVT was divided by list size to give time per patient. The relative time per patient was calculated where the mean time per patient equalled 1. Practices A and C (smaller practices with long childhood appointments and a high proportion of non-clinical time) spent the most time on vaccination activities, whereas practices E and H spent relatively little time overall. Figure 4 shows the proportion of TPVT spent by different staff groups with practice ranked in order of total proportion of time spent by the PN (from 70.8% at practice E to 32.6% at practice H). Practice E had a relatively streamlined and automated administration system, reducing the overall administration time. Practices E, F and B all had relatively large proportions of PN time spent on non-clinical activities, which was primarily due to a significant role in checking the fridges, stock and ordering vaccines. Practice G had a particularly large proportion of administrative time due to receptionist involvement on reminder/recall activities. Practice H’s system of having two PNs and two HCAs in a vaccine clinic significantly reduced the overall amount of time spent by the PN. Overall, the PM had relatively small roles in vaccination, except in A where they were the only member of administrative staff and D where they had a large role in both running searches and submitting data.

### Comparison to service requirements

Table 6 maps the provider requirements from the core service specification and quality standards (QS) recommended by NICE (except QS5 that relates to young offenders) [17, 18]. No practices had a focus on reducing disparities in coverage or interventions to focus on underserved population groups. Similarly, no local communication strategies had been implemented and only practices G and J had patient involvement. A range of contact, reminder and recall systems were used for childhood vaccines; however, for adults, most practices vaccinated only when the patient was attending for another purpose (e.g. health check or for seasonal flu).

**Table 6:** A comparison between implementation of NHS England requirements and NICE quality standards by practices

Domain	Requirement/standard	Adherence by practice
Outcome	2.4: to offer immunisation to 100% of eligible individuals in accordance with guidance.	Childhood: coverage at the large practices G, H and J is much lower than average and well below the 95% target. Adulthood: similarly, coverage at the larger practice (F, G, H and J) is significantly lower than average.
Equity	2.11: to be able to demonstrate what systems are in place to address health inequalities and ensure equity of access to immunisation.	None of the included practices had any specific interventions or services in place to increase uptake in any population or demographic groups with low coverage.
	2.11: to have procedures in place to identify and support those persons who are considered vulnerable/hard-to-reach	None of the included practices had any specific system in place to identify vulnerable or hard-to-reach populations. All practices did follow-up with parents of all young children who did not attend for vaccination.
Service delivery	3.6: to provide core programme elements, as covered in The Green Book.	18 programme elements are described, of which were met by all practices, except reducing variation (none), patient involvement (G and J only) and local communications strategies (nothing, aside from information provision within practice).
	3.10: to address poor uptake for the services where local delivery is lower than the key deliverables to reduce the variation in local levels of performance.	None of the included practices had a system for accessing, evaluating and discussing data relating to their immunisation outcomes or focus on reducing local variation in their local population.
Missed opportunities	3.8: to take every appropriate opportunity to check vaccination status and offer immunisation to individuals who may have missed or not fully completed the national routine schedule. QS2: children and young people identified as having missed a childhood vaccination are offered the outstanding vaccination.	Practices A, E, G and J discussed having a commitment to opportunistic vaccination. However, this was primarily for providing adults with singles and PPV when attending for influenza or other chronic disease health checks. Children were followed up more intensively by all practices at earlier ages, leaving less room for opportunistic vaccination. None of the practices had a specific strategy or protocol for reducing missed opportunities.
Consent	3.9: to adhere to The Green Book guidance on consent.	This was undertaken by all practices.
Assessment	3.10: to have systems in place to assess eligible individuals for suitability by a competent individual prior to each immunisation. QS4: children and young people have their immunisation status checked at specific educational stages.	Aside from the use of searches on computer systems and the general commitment to opportunistic vaccination by some practices (A, E, G and J), no specific protocol or plan was used to check immunisation status. This was especially true for adolescents unless subject to a specific campaign (e.g. meningitis campaign).
Information systems	3.10: assessed the immunisation record of each individual to ensure that all vaccinations are up to date QS3: children and young people receiving a vaccination have it recorded in their GP record and the Child Health Information System (CHIS) and in their personal child health record.	Record keeping was a high priority for all practices, although it was found to be time consuming and complex.
	3.10: systems in place to identify those in clinical risk groups and to optimise access for those in underserved groups	In all practices, the electronic record system was used to identify patients in clinical risk groups, as per the schedule; however, no practices used it to identify people in specific underserved groups.
	3.10: arrangements in place to report and co-ordinate responses to outbreaks of diseases	This was undertaken by all practices.
Reminder, recall	3.10: systems in place to identify, follow up and offer immunisation to eligible individuals 3.10: arrangements in place that enable them to identify and recall under- or unimmunised individuals and to ensure that such individuals are offered immunisation in a timely manner. QS1: children and young people who do not attend their immunisation appointment are followed up with a written recall invitation and a phone call or text message	There was large variation in method and frequency of patient contact, reminder and recall activities. For childhood appointments, all practices sent letters first and used phone calls to follow up non-responders. Practices A and C sometimes called patients first. Practices A, C, H and G also used text messages. All initial patient contacts were made by a receptionist or administrator and follow-up of non-responders to non-attenders was sometimes undertaken by the PN (A, F, G and J). For adults, most practices vaccinated opportunistically when an eligible patient was attending for a check (e.g. diabetes) or for flu vaccine (C, D, E, F, G and J). Practices B and H also sent invite letters. Practice A was alone in phoning older adults.
Vaccine administration	3.12: the provider has a duty to ensure it has, or will have, trained and competent staff to deliver (any) given immunisation programme they agree a contract for	This was undertaken by all practices.
	3.12: the professional lead in the provider organisation must ensure that all staff are legally able to supply and/or administer the vaccine	This was undertaken by all practices.
Storage and wastage	3.13: have effective cold chain and administrative protocols that reduce vaccine wastage to a minimum and reflect national protocols	Responsibilities for maintaining the cold chain was divided between practices who allowed administrative staff to do this (C, G and H) and practices that used the clinical staff (A, B, D, E, F and J).
Ordering	3.14: centrally procured vaccines must be ordered via the ImmForm online ordering system	The distribution of ordering was split similarly to the requirement above.

## Discussion

This is the first study to evaluate how GP practices organise the delivery of the routine vaccine programme in England. We have defined a 14-stage CDVC for vaccination at primary care level alongside a process map. Overall, two thirds (59.7%) of activity was spent on administrative tasks and there was significant variation in allocation of activities between clinical and non-clinical staff, with some models of delivery placing a higher time burden for administration onto nurses. Doctors were largely not involved in vaccination. The mean appointment length for childhood appointments was 2.4 times the length in the practice with the longest appointments (C) than the shortest (H). This was due to significant variation in how appointments were arranged within the practice’s working week, as well as the fixed appointment length (10, 15 or 20 min) on the booking system. The range for adult appointments was narrower but still a more than twofold differential in length (2.1). Total time spent on vaccination relative to practice size varied greatly; however, this was not related to overall performance. Small- to average-sized practices, whether urban (B, C) or rural (A, D), had better performance overall. Greater capacity, as measured by appointments per patient, may be associated with higher coverage in children, but not for adults, and this requires further investigation.

### Fidelity

To further compare and analyse the variation in implementation between the different practices, we have drawn on the concept of fidelity, which is defined as ‘the degree to which programs are implemented as intended by the program developers’, which acts to modify the relationship between the intervention (vaccination) and outcomes (coverage, VPD incidence) [27]. Vaccination is a long-standing complex public health intervention that has been modified over time to reflect an emerging evidence base. Implementation here is defined as ‘a controlled activity aiming to introduce and encourage uptake of a new policy or intervention that embodies pre-defined criteria’ [28]. In this context, the controlled activity is providing the routine vaccination programme and the pre-defined criteria include national evidence-based policy and guidelines. Changes to policy and guidelines are outside the control of the practices and act as interventions with variable penetration. This raises the question of implementation fidelity*:* To explore this further, we have drawn on the conceptual framework developed by Carroll et al. as modified by Pérez et al., which considers the following factors that affect implementation fidelity: adherence (including content and amount (coverage, frequency and duration)), moderators (complexity, facilitation, quality and participant responsiveness) and adaptation [27, 29].

#### Adherence

Adherence is defined as whether the programme is delivered as it has been designed. The use of evidence by practices when delivering the programme was variable depending on the content. For the vaccination programme, there are two relevant elements of content to consider: the clinical and administrative components. For the clinical component (i.e. the schedule), there was very high adherence from all practices for children. However, this was much lower for adult vaccines with six practices only vaccinating when an eligible patient attended for another service.

For the administrative components in both adults and children, there was very variable content adherence, particularly in terms of reminder/recall activities, communications and service design (Table 4). A range of systems was used for contacting patients, most often involving letters and phone calls (in which administrative and reception staff have a large role) with a smaller number of practices using text messages, which have good evidence of effectiveness at increasing coverage [30]. Only two practices used active recall for adult vaccines (G and J), despite evidence for interventions that reminder/recall activities can increase coverage [31]. This creates variability in the amount of intervention delivered to the population (sometimes described as the ‘dose’—e.g. number of reminder letters, availability, frequency and duration of appointments, availability of education materials). This was particularly important for the larger practices with lower coverage, which all spent relatively little time delivering the vaccine programme (F, G, H). In a large, busy practice with no dedicated clinic (G, J), patients may have difficulty in accessing appointment time for vaccination and could have contributed to these practices’ lower coverage. Despite low coverage for childhood (G, H and J) and adult (F, G, H and J) vaccinations, no practices had interventions or services in place to increase uptake in any population or demographic groups with low coverage and none had community information or education plans.

Reducing missed opportunities for vaccination (MOV) features in both the service specification and quality standards, the practices that did report a commitment to opportunistic vaccination (A, E, G and J) did not have a system of reducing MOV. This could potentially be a suitable programme intervention that may increase coverage without requiring significant increases in capacity (such as patient tracking or provider prompts), although evidence is limited for effective interventions [32].

#### Moderators

Organising vaccination is highly complex and thus is likely to have lower implementation fidelity as a result [27]. This in part is because the programme has evolved over time, rather than implemented from scratch, but also because some ability for local tailoring of the administrative content is planned into the guidance [17]. This means that the administrative recommendations are less specific than the clinical recommendations, which is known to reduce likelihood of implementation [33]. Research on NICE guidelines specifically has shown that initially implementation is strategic, but as time passes, it becomes increasingly sporadic and subject to local contextual factors [28].

Similarly, the facilitation strategies (training, support and guidance) are more robust for the clinical elements of the programme. Changes to the vaccine schedule and guidance in The Green Book are followed closely [34], particularly via the vaccine update email from Public Health England [35]. Nurses also undertake annual training, which is primarily clinical. However, there is no equivalent guidance for the non-clinical components to the programme and administrative staff do not receive any training, despite having a significant patient-facing role. Aside from outcome data, this study did not collect any quality metrics from practices, making quality, in terms of patient experience, difficult to assess. This may be a significant modifying factor affecting performance. Of note, the single practice with a low score for friends and family recommendation (F) also had very poor adult vaccination coverage (56.1%), suggesting a quality factor may be important. However, the family and friends test has been criticised as lacking robust rationale and evidence, so further work is required on relevant service quality factors for vaccination.

Participant responsiveness is related to a patient’s view of whether an intervention is useful and relevant to them, thus affecting uptake. All practices reported some experience of having parents and patients decline vaccinations; however, overall, this was described as being a relatively minor problem. However, vaccine hesitancy is an increasing trend in high-income countries and interventions to reduce hesitancy require specific training to implement effectively [36, 37]. More common was persistent non-attenders, who either did not respond to letters or calls or booked appointments and did not attend (DNA). These patients were well known to the practices and often DNA for other appointment types as well. There is little research evidence on how to identify and provide services to improve coverage in persistent non-attending patients.

#### Adaptation

Adaptation is the process of an intervention being changed by an organisation after implementation and may be a critical component for intervention success [29]. The clinical component of the programme shows very little scope for adaptation and is delivered with high fidelity. However, both task allocation and administrative activities show relatively high adaptability, as evidenced by the different organisational structures in place at each of the practices included here. This is significant as people management factors within healthcare organisations, including role descriptions, performance management, stress and leadership, are known to impact organisation effectiveness [38]. It is likely that this adaptation is related to the specific organisational context of each practice, which will be explored in a subsequent analysis of the qualitative data.

### Reducing inequities

There are long-standing inequities in vaccination coverage in many countries, including England and across Europe, as a result of vaccine programmes not providing adequate services to all communities [4, 13, 39–46]. Pockets of under-immunised children and adults are likely one of the factors to have contributed to the rise in VPD incidence and outbreaks seen across Europe in recent years [1, 45]. There is some expectations described in both the service specification and quality standards relating to practices implementing systems to identify and reduce disparities between local population groups. However, there is no clear mechanism for this to be delivered or monitored. Practice staff do not have access to data in sufficient detail to be able to assess local inequities and are unlikely to have the capacity to design and deliver a bespoke intervention without support and guidance. This is despite there being evidence of effectiveness for several categories of interventions to reduce inequities in low-income, ethnically diverse urban settings, such as community-designed multi-component interventions, focussed, escalating reminder-recall activities or alternative service provision models such as home visiting [4]. In practice, the responsibility of identifying and addressing inequities in immunisations is shared among organisations in the public health administration, where local, regional and national public health teams are best placed to identify inequities and suggest evidence-based approaches to addressing them, with general practice implementing these approaches in order to vaccinate those underserved communities. However, since the implementation of the reforms in 2013, data sharing is more complex and the role of regional vaccination managers has changed, reducing their ability to support and evaluate practices’ performance [11]. None of the practices in this sample had experience of working with any external organisation in this way.

### Capacity

A large review of the organisation and implementation of health improvement interventions in primary care identified the strong evidence base for GP practice-led interventions but also noted the increasing complexity of public health services commissioning following the organisation reforms implemented in 2013, including the contracting arrangements for the vaccine programme [47]. Relative time spent delivering the vaccination programme did not appear to have an effect on coverage. Of the two, large practices in London, practice G spent slightly less than average time on vaccination during the study period but had very low rates of coverage overall, and practice J spent roughly average amount of time but also had very low coverage. However, higher capacity for children, in terms of appointments per patient, may be associated with higher coverage. The strength of this association will need to be evaluated with a larger study. While one solution may be to expect these practices to increase the availability or frequency of vaccination appointments, this may not be possible due to the multiple competing demands on staff time at a busy, urban practice with a relatively deprived population with high need, without commensurate increases in funding [9, 48].

### Implications for policy

Mandatory reforms of locally determined systems by national-level bodies are unlikely to be successful given the level of variation between practices. However, information relating to variation in staffing structure and task allocation should be distributed to practices to compare their model and determine if it could be made more efficient, particularly in terms of allocated appointment length. The role of administrators and receptionists was significant, and support in terms of education and training to this group of professionals could improve local service delivery. Task shifting, including support for a greater use of HCAs, could also increase practice capacity by reducing time spent by nurses. Either additional support needs to be given to practices to implement strategies to reduce inequities or this function should be allocated to another branch of the public health system. Rationalisation of the data collection and reporting systems required could also reduce the administrative time burden on practices.

### Strengths and limitations

The strengths of this study include the collection of standardised information, using multiple methods simultaneously, to evaluate implementation of vaccination delivery at a broad range of GP practices in England. This has enabled detailed comparison of GP practice organisation for the first time. Limitations are that this is a small, convenience sample of practices and thus subject to selection bias, making them unlikely to be representative of the population of GP practices at large, especially as practices had higher QOF and recommendation scores than average. No very small or very large practices were included in the sample. The methods rely on self-reporting of activities, which may be subject to reporting bias. Activity logs were kept during different weeks at each practice and activity is not consistent throughout the year. The sample was too small for further statistical analysis to measure association effects.

## Conclusions

There is variation in how GP practices in England implement the delivery of the routine vaccination programme. Areas of greatest variation include the method of reminder and recall activities, structure of vaccination appointments and task allocation between staff groups. Introduction of organised reminder and recall activities for adults could improve coverage. Most (60%) activity was spent on administrative tasks, which had lower fidelity of implementation to guidelines and standards. Implementation of clinical activities had very high fidelity. Appointment length and time spent on vaccination did not appear to be related to coverage; however, capacity in terms of availability of appointments per patient could be related and requires further investigation. Further work is also required to evaluate other contextual factors, including the working culture within practices.

## Appendix

### Topic guide

**Table 7:** A modified version of the below guide was used if the interview only had administrative staff

Topic	Prompt	Notes and possible questions
Welcome and introduction	Intro	- Background to the project - My role and LSHTM
	Scope of interview	- Overview of purpose - Confidentiality - Topics to be covered - Time for questions - Confirm consent
Organisation and role	Intro	This section is looking to understand how the routine vaccination programme is organised within your GP practice.
	Role and responsibility	- Please describe your role within the practice team in delivering the vaccination programme. - How long have you been in this position for? - How much of your working time do you spend on vaccination per week?
	Vaccination programme organisation	- How is the vaccination programme organised within your GP practice? - Which other staff members are involved?
Inputs	Intro	- This section is looking to understand how the practice uses information to make decisions about how to run the programme.
	Information sources and use	- What information sources do you use? - How are you informed of changes to the programme? - How often to you undertake training?
	Data knowledge and management	- Where do you get information from relating to your practice’s performance? - How often do you receive updates about coverage levels?
	Resources (financial and human)	- Do you have responsibility for the financial management of the programme? - Do you know how the programme is funded? - Is the funding adequate to provide the programme? - What do you think about the current system of payments for the vaccination programme?
	Networks	- Are you a member of any local or national networks relating to vaccination? - What organisations do you interact with locally related to vaccination? - What information do you receive from these sources? - Do the networks provide any other benefits? - Which other external organisations (e.g. schools, local government) do you interact with regularly?
Sensemaking	Intro	This section is seeking to understand how vaccination is perceived and prioritised within your practice.
	Leadership	- Is there an identified person who leads the programme? - If the leadership role is split, please describe how this works? - Is leadership important for delivery of the programme in your practice?
	Decision-making	- Who is responsible for making decision relating to the programme? - Do you have a regular practice meeting to discuss vaccination? How often? Who attends? - Is there a local or regional committee or board that meets? How often? Who attends? - How often do you make changes in how you deliver the programme?
	Climate and culture	- How much of a priority is vaccination when compared to other areas? - Are staff members and the management supportive of vaccination? - Could the way your practice organises the programme be improved? If so, what are the barriers?
	Interpersonal relationships	- Do staff work together well to deliver the programme? - Do staff interact with external organisations well? - Are there any interpersonal barriers to running the programme?
	Responses to change	- How well does your practice respond to changes in the programme? - How long does it take changes to the programme to be implemented?
Activities and outputs	Intro	This section is seeking to understand what vaccination activities you undertake and the uptake of these.
	Task allocation	- How are roles and responsibilities distributed? - Is it always clear who is supposed to be doing what?
	Time allocation	- How much time is dedicated to vaccination? - Is this enough? Or too much/little?
	Systems and processes	- Is the way the programme organised suitable? - Is the system of delivering vaccinations clear to all staff? - Could it be improved? - How are incidents reported?
	Data collection and submission	- How are data collected and submitted? - What is your role in this process? - Is it done well? Or could it be improved?
	Uptake and access	- Is there good uptake of vaccination in your practice? - Do you think patients have any problems accessing services?
	Interventions	- Are you involved with any interventions to improve access/uptake? If so, please describe. - If no, do you think there would be any role for an intervention at your practice? If so, for what purpose?
	Workload and capacity	- How do you find the workload of running the programme? - Do your colleagues feel the same way? - Do you have capacity to increase uptake of the programme?
Outcomes	Intro	This section is seeking to understand what the overall outcomes of the programme are in your practice.
	Patient factors and perception	- Do you think the type of population you service makes any difference to the coverage levels you achieve? - Do your patients hold any views that affect vaccination coverage?
	Community relationship	- Is the practice integrated into the local community? - How do you feel about the patients in your local area?
	Coverage levels and performance	- Do you know how well your practice performs? - What indicators do you use? - Do you feel this could be improved? If so, how?
Wrap up	Open space for discussion and questions	- Has everything been covered? - Space for any other questions/issues to be raised/discussed.

## Abbreviations

AD: Administrator
APM: Assistant practice manager
CDVC: Care delivery value chain
CHIS: Child Health Information System
CI: Confidence interval
CRN: Clinical Research Network
DH: Department of Health
DTaP/IPV/Hib: Diphtheria, tetanus, pertussis, polio, *haemophilus influenza type b* vaccine
GP: General practitioner
HCA: Healthcare assistant
Hep B: Hepatitis B vaccine
Hib: *Haemophilus influenza type b* vaccine
HPV: Human papillomavirus
HSCA: Health and Social Care Act
Men ACWY: Meningococcal groups A, C, W and Y
Men B: Meningococcal group B
Men C: Meningococcal group C
MMR: Measles, mumps and rubella
MOV: Missed opportunities for vaccination
NHSD: National Health Service Digital
NHSE: National Health Service England
NICE: National Institute for Health and Care Excellence
PCV: Pneumococcal conjugate vaccine
PHE: Public Health England
PM: Practice manager
PN: Practice nurse
PPV: Pneumococcal polysaccharide vaccine
QOF: Quality Outcomes Framework
QS: Quality standard
R: Receptionist
SMS: Short message service (text message)
TDABC: Time-Driven Activity-Based Costing
TPVT: Total practice vaccination time
VPD: Vaccine preventable disease
